# The production and recognition of psychiatric original articles published in languages other than English

**DOI:** 10.1186/1471-244X-13-102

**Published:** 2013-03-26

**Authors:** Christopher Baethge

**Affiliations:** 1 Department of Psychiatry and Psychotherapy, Cologne University Medical School, Kerpener Straße 62, Cologne, 50937, Germany; 2 Deutsches Ärzteblatt, Deutsches Ärzteblatt International, Cologne, Germany

**Keywords:** Journals as topic, Bibliometric analysis, Psychiatry

## Abstract

**Background:**

Whereas the most influential journals in psychiatry are English language journals, periodicals published in other languages serve an important purpose for local communities of clinicians and researchers. This study aimed at analyzing the scientific production and the recognition of non-English general psychiatry journals.

**Methods:**

In a cohort study, the 2009 volume of ten journals from Brazil (1), German language countries (5), France (2), Italy (1), and Poland (1) was searched for original articles. Patterns of citations to these articles during 2010 and 2011 as documented in Web of Science were analyzed.

**Results:**

The journals published 199 original articles (range: 4–46), mostly observational studies. Half of the papers were cited in the following two years. There were 246 citations received, or an average of 1.25 cites per article (range: 0.25-4.04). Many of these citations came from the local community, that is, from the same authors and journals. Citations by other periodicals and other authors accounted for 36% [95%-CI: 30%-42%], citations in English sources for 33% [28%-39%] of all quotations. There was considerable heterogeneity with regard to citations received among the ten journals investigated.

**Conclusion:**

Non-English language general psychiatry journals contribute substantially to the body of research. However, recognition, and in particular recognition by the international research community is moderate.

## Background

Over the last six decades psychiatry has undergone a profound shift to English as the main language of communication [1]. This period has witnessed switches to English by many important journals that originally had been published in other languages, for example *Acta Psychiatrica Scandinavica* or *European Archives of Psychiatry and Clinical Neuroscience*. Today, all major journals publish in English, and there is no non-English journal among the first 75 psychiatric periodicals in the Journal Citation Report. The situation in psychiatry only reflects the situation in medicine in general: the overwhelming majority of journals indexed in Medline are in English, and at least nine out of ten newly indexed sources use the lingua franca [2].

At the same time, the number of journals publishing in languages other than English is rapidly increasing, partly due to fast growing research communities in, for example, Brazil, China, India, or Russia [3]. Also, doctors express the wish to be provided with scientific articles in their native language. For example, in a survey of German psychiatrists, about 70% considered it important that educational material is presented in their mother tongue [4]. In addition, it has been shown that doctors in Scandinavia remembered more after reading an article in their native language in comparison to the same material in English [5]. Therefore, journals in local languages serve an important purpose.

Whereas there are no official figures regarding the number of German language medical periodicals, the German central library of medicine in Cologne subscribes to no less than 1200 periodicals in German. There seems to be a core of high level English language journals and a corona of many journals published in national languages [2].

In an earlier study [6], we found that there is a lively scene of national language psychiatry journals in Germany: In 2009, there were 74 journals that addressed psychiatrists – solely or among other groups. Ten of those periodicals were general psychiatry journals that, in total, had published almost 400 review articles, 56 case reports but also almost 100 original articles. Since some of the original articles addressed other specialties (e.g., neurologists), we counted 79 psychiatric original articles. The majority (72) were published in those five journals that were listed in Thomson Reuter’s Journal Citation Reports (that is, journals that had an impact factor).

The importance of cultivating national languages has been emphasized [2], but it has also been argued that publishing original work is worthwhile only as long as it is in English [7]. However, there is a scarcity of data that may inform such debates, and the extent to which non English original articles are recognized in the research community is unknown. Citations are an accepted proxy for scientific recognition. Therefore, they seem to be ideally suited for an investigation into the recognition, the “impact”, of scientific work.

Accordingly, an analysis of citations to original articles published in ten general psychiatric journals published in languages other than English was carried out. Specific aims were to find out how many articles were cited, how often they were referred to and by whom.

## Methods

This is a retrospective investigation of a cohort of original articles appearing in 2009 in general psychiatric journals that were published in languages other than English. The present study builds on earlier work on German language psychiatry journals [6]. From this study all 72 psychiatric original articles in German that had been published in the five impact factor journals included were selected for citation analysis in Web of Science.

As a control group a sample of general psychiatry journals publishing in languages other than English and German was selected from Science Citation Index’ psychiatry list. Apart from language and scope (general psychiatry as opposed to, e.g., psychopharmacology or psychiatry of old age or childhood) journals were included when they were available in the German library of medicine (ZBMed, Deutsche Zentralbibliothek für Medizin, Köln) or when they were freely accessible online. Table 1 documents the journals selected. The documentation includes its publishing house, the journal's status regarding to Medline coverage, and both its Impact factor and Article influence score (as reported by JCR 2012).

**Table 1:** Journals included five German language and five international non-English general psychiatry journals selected for citation analysis of original articles

**Journal**	**Language**	**Country of origin**	**Publisher**	**Indexed in Medline**	**IF 2010**	**AI 2010**
Fortschr Neurol Psychiatr	German	Germany	Thieme-Verlag	Yes	0.652	0.095
Nervenarzt	German	Germany	Springer-Verlag	Yes	0.729	0.114
Nervenheilkunde	German	Germany	Schattauer-Verlag	No	0.368	0.033
Psychiatr Prax	German	Germany	Thieme-Verlag	Yes	2.204	0.110
Z Psychiatr, Psychol Psychother	German	Switzerland	Huber-Verlag	No	1.952	0.163
Ann Med Psychol	French	France	Masson Editeur	No	0.161	0.035
Encephale	French	France	Masson Editeur	Yes	0.421	0.094
Psychiatr Pol	Polish	Poland	Wydawniczy Polskiego Torwarzystwa	Yes	0.173	0.051
Rev Psiq Clin	Portuguese^1^	Brazil	Sao Paulo University	No	0.648	0.202
Riv Psichiatr	Italian	Italy	Pensiero Scientifico Editor	Yes	0.207	0.035

^1 ^In 2009 Revista de Psiquiatria Clinica published a limited number of original articles in English and not in Portuguese. Those articles were not included in the present analysis.

Fortschr Neurol Psychiatr, Fortschritte der Neurologie und Psychiatrie; Nervenarzt, Der Nervenarzt; Nervenheilkunde, Nervenheilkunde; Psychiatr Prax, Psychiatrische Praxis, Z Psychiatr, Psychol Psychother, Zeitschrift für Psychiatrie, Psychologie und Psychotherapie.

Ann Med Psychol, Annales Medico-Psychologiques; Encephale, L’Encephale; Psychiatr Pol, Psychiatria Polska; Rev Psyq Clin, Revista de Psiquiatria Clinica; Riv Psichiatr, Rivista di Psichiatria.

IF, Impact factor; AI, Article influence score. The article influence score is different from the impact factor because it does not consider self-cites and does not rely on citations from journals included in Thomson Reuters’ Science Citation Indices only. Similar to Google’s page rank procedure citations are weighted according to the estimated importance of their source. The article influence score is weighted for the number of articles published in a journal (http://www.eigenfactor.org). Impact factors and article influence scores were retrieved from Thomson Reuters’ Journal Citation Report. Of note, both indices take not only into account original articles but all citeable articles of a journal.

Of all periodicals so selected the complete volume of 2009 was screened for original articles. Original articles published in English, as in some cases in Revista de Psiquiatria Clinica, were not included. An original article was defined as a paper presenting new research results. Such papers were selected regardless of the section in which a journal presented an article (original research, reviews, letter to the editors). As a consequence, but rarely, articles were selected even if they appeared in the letters to the editor section because some journals use this section for brief original contributions. Case reports, however, were not considered original articles. They often serve educational purposes and, in general, are not the result of a research project.

Citations to original articles (“received citations”) were included when the citing article was published in 2010 and 2011 and citations were documented in Web of Science. In March and May 2012 for every original article a “cited reference search” was carried out in Web of Science.

Every received citation was characterized with regard to the following criteria: year of publication of the citing article (2010 vs. 2011), author of citing article (self-cite vs. other), language of citing author (same language vs. English), journal of citing article (self-cite vs. other), and language of journal of citing article (same language vs. English). In order to determine the language of citing authors the address of the author, as presented in Web of Science, was used as a proxy measure. Similar to other studies [8] a citation received was rated as coming from an author of the same language when one contributor of an article’s author team fulfilled the criterion. For example, if a French article would be cited in another article with at least one author with an address in France this citation would be counted as coming from the same language. The same definition applied to the status of self-citation: at least one author from the author group of the cited paper in the byline of the citing paper.

The category English includes rare cases of citations received in other languages: not in English and not in the language of the original article (those cases are indicated in Tables 2 and 3). The language status of the citing journal was assessed as defined by Web of Science. In cases of doubt, PubMed, the homepage of the journal, or the full texts of the citing articles were used.

**Table 2:** Cites in 2010/11 to original articles published in five German language general psychiatry journals in 2009

	**Fortschr Neurol Psychiatr**	**Nervenarzt**	**Nervenheilkunde**	**Psychiatr Prax**	**Z Psychiatr, Psychol Psychother**	**Total**
Original articles published in 2009	11	16	13	28	4	72
Cited (in 2010+11) original articles in %, (n)	64% (7)	50% (8)	31% (4)	100% (28)	100% (4)	71% (51)
Cites, (cites per original article)	**14 (1,27)**	**18 (1,13)**	**6 (0,46)**	**113 (4,04)**	**16 (4)**	167 (2,32)
…thereof:						
in 2010	**43% **(6)	**33% **(6)	**83% **(5)	**65% **(74)	**63% **(10)	60% (101)
in 2011	**57% **(8)	**67% **(12)	**17% **(1)	**35% **(39)	**37% **(6)	40% (66)
in the same journal	**27% **(3)	**6% **(1)	**23% **(3)	**58% **(66)	**19% **(3)	46% (76)
in same language journals^1^	**55% **(6)	**17% **(3)	**100% **(6)	**86% **(97)	**100% **(16)	77% (128)
in English	**73% **(8)	**83% **(15)	**0% **(0)	**14% **(16)^**2**^	**0% **(0)	23% (39)
by the same authors	**71% **(10)	**28% **(5)	**33% **(2)	**16% **(18)	**81% **(13)	29% (48)
by authors of the same language	**93% **(13)	**39% **(7)	**100% **(6)	**95% **(107)	**100% **(16)^**3**^	89% (149)
by other authors + journals	**27% **(3)	**68% **(12)	**33% **(2)	**30% **(34)^**4**^	**19% **(3)	32% (54)
by other authors + in English	**27% **(3)	**56% **(10)	**0% **(0)	**11% **(12)	**0% **(0)	15% (25)

^**1 **^includes same journal; ^**2**^English includes two cites in non-English articles (1 in French, 1 in Italian); ^**3 **^15/16 cites are linked to the prolific editor of Z Psychiatr, Psychol Psychother as author of citing articles ^**4 **^If articles were taken into account that seem to have been published, at least in part, out of bibliometric interest or ambition [9,10] this number would shrink by 13 to 21 (reducing the rate of cites by other authors and journals to 19%).

Fortschr Neurol Psychiatr, Fortschritte der Neurologie und Psychiatrie; Nervenarzt, Der Nervenarzt; Nervenheilkunde, Nervenheilkunde; Psychiatr Prax, Psychiatrische Praxis, Z Psychiatr, Psychol Psychother, Zeitschrift für Psychiatrie, Psychologie und Psychotherapie.

**Table 3:** Cites in 2010/11 to original articles published in five international non-English general psychiatry journals in 2009 (publishing in French, Polish, Portuguese, and Italian)

	**Ann Med Psychol**	**Encephale**	**Psychiatr Pol**	**Rev Psyq Clin**	**Riv Psichiatr**	**Total**
Original articles published in 2009	46	37	17	7	20	127
Cited (in 2010+11) original articles	30% (14)	54% (20)	35% (6)	71% (5)	25% (5)	39% (50)
Cites	**25 (0,54)**	**35 (0,95)**	**6 (0,35)**	**8 (1,14)**	**5 (0,25)**	**79 (0,62)**
…thereof:						
in 2010	**48% **(12)	**26% **(9)	**33% **(2)	**50% **(4)	**20% **(1)	**35%**(28)
in 2011	**52% **(13)	**74% **(26)	**68% **(4)	**50% **(4)	**80% **(4)	**65% **(51)
in the same journal	**32% **(8)	**14% **(5)	**33% **(2)	**25% **(2)	**100% **(5)	**28% **(22)
in same language journals	**60% **(15)	**31% **(11)	**33% **(2)	**38% **(3)	**100% **(5)	**46% **(36)
in English^1^	**40% **(10)	**69% **(24)	**68% **(4)	**63% **(5)	**0% **(0)	**54% **(43)
by the same authors	**60% **(15)	**31% **(11)	**33% **(2)	**38% **(3)	**20% **(1)	**39% **(31)
by authors of the same language	**88% **(22)	**57% **(20)	**50% **(3)	**63% **(5)	**80% **(5)	**70% **(55)
by other authors + journals	**28% **(7)	**60% **(21)	**68% **(4)	**38% **(3)	**0% **(0)	**44% **(35)
by other authors + in English	**20% **(5)	**48% **(16)	**68% **(4)	**63% **(3)	**0% **(0)	**35% **(28)

^1 ^English includes two cites in non-English articles (1 in Rumanian, 1 in German).

Ann Med Psychol, Annales Medico-psychologiques (language of publication: French); Encephale, L’Encephale (French), Psychiatr Pol, Psychiatria Polska (Polish), Rev Psyq Clin, Revista de Psyquiatria Clinica (Portuguese; not considered were original articles in English published by Revista de Psyquiatria Clinica in 2009 and original articles from issue 2 in 2009 because they seem not to be covered in Web of Science; both factors render the number of original articles included small); Riv Psichiatr, Rivista di Psichiatria (Italian).

Findings are presented as raw numbers, percent, confidence intervals, or ratios where appropriate. Dichotomous outcomes were compared using a χ^2^-test.

## Results

The ten journals included in this study published 199 original articles in 2009. The dominant type of study was observational, often aiming at describing psychopathological, demographic and clinical characteristics of patients. Biological studies and randomized controlled trials were almost completely absent in this cohort of publications. In 2010 and 2011 half of all papers (n=101) were cited in journals included in Web of Science, with the total number of received cites reaching 246 (range 1–17 cites per article). Thus, an original article was cited, on average, 1.25 times, 0.668 times in 2010 and 0.588 in the 2011.

Of the 246 received citations, 98 were from the same journal (40%, 95%-CI: 34%-46%). In all, two out of three citations received (164/246, 67%, 95%-CI: 61%-72%) appeared in periodicals publishing in the same language. Accordingly, 33% (82/264, 95%-CI: 28%-39%) originated with journals in English.

Roughly one third of the citations received were created by the authors of the original articles themselves (79/246, 32%, 95-CI: 27%-38%). Eighty-three percent (204/246, 95-CI: 78%-87%) were found in articles with at least one author of the same language indicating that in 17% (95-CI: 13%-22%) the original articles were referred to by authors or author teams of different nationality or language.

Taken together, thirty-six percent (89/246, 95%-CI: 30%-42%) of the original articles were cited by other authors as well as other journals, and about one in five (53/246, 22%, 95-CI: 17%-27%) of all cites to the original work in non-English journals came from other author groups that had published in English.

The journals differed considerably with regard to all measures analyzed in this study. For example, the number of original articles published in 2009 ranged from 4 (Z Psychiatr, Psychol Psychoth) to 46 (Ann Med Psychol). The lowest number of cites to those papers in 2010/11 was, at the journal level, 5 (Riv Psichiatr) whereas the highest number of citations received per journal amounted to 113 (Psychiatr Prax). The number of citations received per original article ranged from 0.25 to 4.04. Similar variations can be found regarding the proportion of journal self-citation (range: 6-58%), the proportion of self-citations by the authors of the original article themselves (16%-81%), the percentage of citations that came from other authors and journals (0%-68%), and regarding the category of cites most distant from the original publication: references in English language journals by other authors (0-68%), with two journals without English citations by other authors.

When the five national language journals from Brazil, France (2×), Italy, and Poland were compared to German language journals in a group-wise fashion no meaningful differences between the groups emerged, with three notable exceptions: Firstly, they published, as a group, more original articles than the German journals (127 vs. 72). Secondly, the number of citations received was higher in the group of German language psychiatric periodicals (167 vs. 79; cites per article: 2.32 vs. 0.62). Thirdly, the proportion of received citations other than self-citations by journals or authors is higher in the comparison group (44% vs. 32%) - a result even more pronounced when only external English citations (without self-cites by authors) were considered: 35% vs. 15% (95-CI: 26%-47% vs. 10%-21%; χ^2^-test: p<0.001). Still, in absolute numbers, an original article from German journals had a higher chance of being cited by other authors in English language journals (0.35 vs. 0.22) (Tables 2 and 3).

Journals not indexed in Medline (Nervenheilkunde, Z Psychiatr, Psychol Psychother, Ann Med Psychol, Rev Psiq Clin; see Table 1) were less often cited than those covered in this database: 55 citations to 70 arcticles published in the former group vs. 191 citations to 129 original papers in the latter (0.79 vs. 1.58 cites per article). This result did not change meaningfully when only citations from other authors and other journals were taken into account (0.21 vs. 0.57 cites per article).

## Discussion

This study yielded several findings: Firstly, regardless of their educational focus, general psychiatry journals published in languages other than English contribute significantly to the body of research: The ten periodicals under study alone published 199 original articles in 2009. Secondly, the average number of citations received in 2010 and 2011 was 1.25. This figure decreased to 0.45 for citations received by other authors and other journals (external citations), and to 0.27 for citations received by other authors and in English (external international citations). Thirdly, there are considerable differences among the journals included. Therefore, non-English general psychiatry journals do not share a distinct pattern with regard to the reception of their original articles.

In an earlier study, we found that in 2009, decades after English became established as the universal language of medicine, general psychiatry journals in German language focused on review articles and educational material but still published a substantial number of original articles [6]. The present analysis suggests that this applies also to the Brazilian, French, Italian, and Polish journals included in this study. Some of the roughly 200 original articles published in the ten periodicals scrutinized dealt with specifically regional topics such as testing of psychometric scales in the national language or research regarding local health service systems. And yet, many of the papers presented observational studies that were, in the reading of the author, similar to those appearing in many English language periodicals regarding both scope and quality. Therefore, there is no reason to believe that the topics in regional language psychiatric journals are different from English language journals. However, much of what by some in the field is considered high-level psychiatric research, e.g., large scale epidemiological work, biological studies, or RCTs was almost completely absent from this sample of non-English sources in 2009.

What scientific reception does research published in non-English journals enjoy? Judged by citations received in the two years following publication, it seems that only a minority of articles reach a wider audience. For seven out of ten journals, fewer than half of all received citations were external in the narrow definition of this study, that is, they came from other authors and journals. And in six of those periodicals the proportion was a third or less (total for all ten periodicals: 36% of citations). With regard to jumping the language barrier the situation is even more pronounced: Citations received by other authors and in English language journals, scientifically probably the most highly valued citations, accounted for only 22% of all received citations indicating that it is unlikely for a research paper in one of the languages investigated to be picked up by international authors. The original articles from four journals were not cited in English sources at all. Besides proportions, absolute numbers of external (89/249) and international external citations (53/249) for this sample of original articles were moderate: A virtual impact factor reflecting the international citations to this sample of original articles over the observation period 2010/11 (without self-citations) would be 0.27 (53 cites/199 articles).

Limited recognition of scientific work outside the group of authors of the same journal may have different reasons. Obvious grounds include the inability of other scientists to read the article or the poor quality of the research. Within the sphere of a given language, however, it is also possible that the community of researchers and clinicians interested in the topic may be very small and well covered by the publishing journal. However, slight differences in scope notwithstanding, the journals included in this study were general psychiatry journals covering broad and overlapping areas of research. Still, for certain projects this explanation may have some merit, for example in health services research, a topic often restricted to the local health system. In addition, differences in the countries from which the journals originate cannot be dismissed: For example, the number of Polish psychiatry journals is smaller than that of French or German periodicals.

The moderate rate of external citations to the original articles under study may also be an artifact of high self-citation rates. In general, self-citation rates are substantial, both at the level of journals (total: 40%) and authors (total: 32%). Of note, the differences between the journals included are considerable, with ranges of 6%-100% (journals) and 16%-81% (authors), respectively. However, Aksnes, studying self-citation by Norwegian authors on the basis of approximately 1300 papers in psychiatry and psychology, found an author self-citation rate of 21% over the whole observation period of, on average, 11.6 years [11]. Self-citation, however, declined over time indicating that a two-year self-citation rate of about one third is not restricted to the present set of journals. Studies of three top general medicine journals and of eight general orthopedic journals showed lower figures (6.5% and 1-15%, respectively [8,12]), but all of those journals appeared in English.

There are many reasons to cite one’s own work or the work that has been published in the same journal, many of them perfectly understandable. Often, for example, self-cites refer readers to important related articles, to sources of information presented in the text, or to papers that are essential for understanding the historical development of research projects. Other reasons include inflating author or journal citations as an attempt at improving evaluation statistics such as Impact factor or Hirsch-Index. Apart from obviously false citations it is difficult to find a universally accepted definition or even a numerical cut-off value for the unscientific use of self-cites. Eugene Garfield and Thomson Reuters as the inventor and publisher of the Impact factor have taken a quite liberal stance [13], but the Journal Citation Reports provide self-citation rates. New citation statistics such as the Eigenfactor, or its derivative, the Article influence score (Table 1) exclude self-cites. Still, there are other ways of artificially inflating the IF of journals, for example, publishing (in other periodicals) papers replete with references to a certain journal, with many citations coming from the last two years, sometimes under the pretext of more or less scientific questions.

Journals from countries outside the anglophone world have reacted differently to the universal shift to English: Some, like most of those presented here, retained their language and are thus important sources for the regional community of clinicians and researchers. The quintessential example is *Annales Médico-psychologiques*, founded in 1843 and notably the oldest still running psychiatric periodical in the world.

Others have switched their publication language to English. Prominent examples include the *European Archives of Psychiatry and Clinical Neuroscience* (founded by Griesinger in 1868, and until 1983 *Archiv für Psychiatrie und Nervenkrankheiten*) and *Acta Psychiatrica Scandinavica* (completely in English since 1973). Today, they have substantially higher Impact factors (3.5 and 4.2, resp. in Journal Citation Reports 2011) than the journals in our sample. It remains unclear, however, whether the impact of those journals on practicing and even on research psychiatrists is as large as the influence of a widely read regional journal like the *Annales Médico-psychologiques*.

A third group of journals publishes bilingually, for example, *Actas Espanolas de Psiquiatria* or *Turk Psikyiatri Dergisi* thereby serving both the interests of the local audience and of the authors, but at the disadvantage of high costs. A particularly striking example of a journal publishing in more than one language is *World Psychiatry*, the official journal of the World Psychiatric Association, that appears in no less than seven languages (Arabic, Chinese, English, French, Russian, Spanish, Turkish).

Finally, some periodicals publish certain articles in their regional language and others in English – in our sample this applies to *Revista de Psiquiatria Clinica* from Brazil*,* and recently also to *Fortschritte der Neurologie und Psychiatrie*[14] as well as to *Nervenarzt*[15], both from Germany.

The limitations of this study should be acknowledged: It has to be borne in mind that citations in journals not covered in Web of Science, citations in books or in doctoral theses, and citations after more than two years were not counted. Therefore, in the strict sense, the results presented here only refer to the observation period and to Web of Science. Other, more inclusive databases may have provided different results: It has been shown that Google Scholar retrieves more citations [16]. However, this comes at the price of lower citation accuracy. There is also a, smaller, difference between Scopus and Web of Science in the number of retrieved citations. Web of Science, however, was chosen because it is the source of the Impact factor, arguably the most important evaluation metric in medicine. Also, Journal Citation Report Science Edition’s subject category Psychiatry alone lists no less than 130 periodicals, not to mention the total of roughly 10.000 journals – quite a few in related subject categories – that are included in the Journal Citation Report. Comparability to the Impact factor was also the motive for choosing an observation period of two years: For the two-year Impact factor articles published in 2009 were evaluated in 2010 and 2011. However, it is likely that with a longer observation period the rate of self-citations would have decreased. Other important figures, such as the rate of international external citations are unlikely to be affected by the observation period. Finally, since the journals included are a convenience sample they are not necessarily typical for all non-English journals in the field. They represent, however, a sizable part of all local general psychiatry periodicals.

## Conclusion

The notable differences among the journals included in this study preclude general statements as to a typical pattern of original article publication and citation. However, even though only a limited set of psychiatric journals was investigated, it is fair to conclude that a non-negligible part of research in countries outside the Anglophone world is still published in languages other than English. During the first two years after publication, the reception of this scientific work, in many cases, is largely restricted to regional research communities, sometimes even to the same journal. International citations are rare. This, however, does not mean that journals publishing in languages other than English do not serve an important purpose for their community.

## Competing interest

Christopher Baethge is editor of Deutsches Ärzteblatt and of Deutsches Ärzteblatt International, a bilingual (German, English) general medical journal.

## Pre-publication history

The pre-publication history for this paper can be accessed here:

http://www.biomedcentral.com/1471-244X/13/102/prepub
